# Spatial and Sex-Specific Growth Variations of Migratory *Coilia nasus* in the Middle and Lower Yangtze, China

**DOI:** 10.3390/biology14091211

**Published:** 2025-09-07

**Authors:** Hongyi Guo, Xuguang Zhang, Wenqiao Tang, Kai Liu

**Affiliations:** 1 College of Fisheries and Life Science, Shanghai Ocean University, Shanghai 201306, China; 2 Engineering Technology Research Center of Marine Ranching, Shanghai Ocean University, Shanghai 201306, China; 3 Shanghai Universities Key Laboratory of Marine Animal Taxonomy and Evolution, Shanghai Ocean University, Shanghai 201306, China; 4 Key Laboratory of Freshwater Fisheries and Germplasm Resources Utilization, Ministry of Agriculture and Rural Affairs, Freshwater Fisheries Research Center, Chinese Academy of Fishery Sciences, Wuxi 214081, China

**Keywords:** anadromous fish, growth modeling, sexual dimorphism, size-dependent migration, Yangtze River conservation

## Abstract

The Japanese grenadier anchovy (*Coilia nasus*), an economically vital anadromous species in the Yangtze River, has experienced severe population decline, prompting a 10-year fishing moratorium. Comprehensive data on its population structure and spatial growth variations during this critical recovery period, however, remain scarce. This study addresses this gap by analyzing 1119 individuals sampled from four sites along the species’ migratory corridor during the 2024 spawning season. We assessed key life-history traits to quantify spatial and sex-specific dynamics. We found significant sexual dimorphism, with females attaining greater size and age than males. The population was highly structured spatially: larger individuals were captured farther upstream, and initially female-biased sex ratios became more balanced inland. Growth modeling revealed that females had higher asymptotic lengths (*L*_∞_) but lower growth coefficients (*k*). Furthermore, macroscopic assessment of gonadal maturity identified contrasting reproductive schedules between sexes along the migratory route. These results demonstrate clear sex-specific life-history strategies and a strong spatial segregation by size and reproductive state. This study provides crucial baseline data from the moratorium period, underscoring the necessity of spatially explicit management—particularly the protection of upstream spawning habitats for larger, more fecund individuals—to aid the recovery of this endangered species.

## Simple Summary

The Japanese grenadier anchovy (*Coilia nasus*), a valuable anadromous fish that migrates up the Yangtze River to reproduce, is endangered due to severe overfishing. To aid its recovery, a comprehensive 10-year fishing moratorium is now in effect. To assess the population’s status during this critical period, we studied over 1100 individuals along their 2024 spawning migration route. Our research revealed significant differences between the sexes: females grow larger and live longer, likely a strategy to maximize reproductive output, while males mature earlier. Crucially, we discovered a clear pattern of size-dependent migration, where larger fish travel farther upstream to spawn. These findings underscore the importance of protecting upstream spawning habitats, as they support larger, more mature individuals. This study provides important baseline data for conservation managers to evaluate the effectiveness of the fishing moratorium and inform this culturally significant species’ recovery efforts.

## 1. Introduction

*Coilia nasus* Temminck & Schlegel, 1846 (Japanese grenadier anchovy), a commercially valuable anadromous fish species, is widely distributed throughout the Northwest Pacific Ocean, predominantly inhabiting coastal waters of China, Japan, and Korea [1,2,3]. In the Yangtze River Basin, this species exhibits remarkable life-history diversity, with the anadromous form historically representing one of the most economically important fishes in the region and celebrated as one of the “Three Yangtze Delicacies” alongside Reeves shad (*Tenualosa reevesii*) and obscure pufferfish (*Takifugu obscurus*) [4].

The life history of anadromous *C. nasus*, characterized by elongated supramaxillary bones, includes extensive spawning migrations from February to September, with clear timing variations between early- and late-running populations [5]. During these spawning seasons, individuals undergo rapid gonadal development and reproduce annually in both lacustrine and riverine systems [6]. Remarkably, some individuals migrate as far as Dongting Lake, a distance exceeding 1000 km upstream for spawning, with both adults and offspring returning to the sea afterward [7]. Such extensive migrations across diverse environmental gradients make *C. nasus* an ideal model for studying spatial variation in fish life-history traits.

Spatial variation in fish life-history traits, particularly growth patterns, represents a fundamental ecological phenomenon driven by environmental gradients such as temperature, food availability, and population density [8]. For anadromous species that traverse diverse habitats from marine to freshwater ecosystems, such spatial heterogeneity can lead to distinct population-specific growth patterns and adaptive responses. Furthermore, sex-specific differences in growth (sexual growth dimorphism) are widespread among fishes and are often linked to differential reproductive strategies [9]. Typically, the sex that invests more heavily in gamete production or parental care may exhibit slower somatic growth, reflecting a fundamental trade-off in energy allocation between growth and reproduction [10]. Understanding these variations is therefore critical for accurately assessing population dynamics and formulating effective management strategies for migratory species.

However, increasing consumer demand has led to severe overexploitation of *C. nasus*, with annual production declining dramatically from 3750 tonnes in 1973 [11] to merely 57.5 tonnes by 2012 [4]. This decline has resulted from multiple anthropogenic pressures, including overfishing, habitat degradation, and environmental stressors [12]. Consequently, *C. nasus* was classified as Endangered on the International Union for Conservation of Nature’s (IUCN) Red List of Threatened Species in 2018. To address these challenges, China implemented a commercial fishing ban for *C. nasus* in 2018, followed by a comprehensive 10-year fishing moratorium across the Yangtze River in 2021 that was aimed at facilitating wild stock recovery [4]. These conservation efforts highlight the urgent need for comprehensive population assessments to evaluate recovery progress.

Scientific investigations of *C. nasus* have evolved significantly over the past decades. Extensive surveys in the 1970s focused on the species’ resources and biological attributes in the Yangtze River [13]. By the 2000s, research emphasis had shifted toward populations in the lower reaches [14]. More recently, particularly following the implementation of the 10-year fishing moratorium, studies have predominantly involved single-site investigations [15,16]. However, a significant knowledge gap remains regarding multi-site investigations spanning from the estuary to the middle reaches, thus limiting our comprehensive understanding of spatial population dynamics and the growth variations predicted by ecological theory.

To address this knowledge gap, our study provides the first basin-scale assessment of *C. nasus* population structure, growth, and reproductive strategies during the critical spawning migration season. Our specific objectives were to: (1) elucidate population structure by analyzing sex ratio and age composition across sampling sites, using scales and otoliths as reliable chronological recorders in fishes [17] for precise age determination; (2) investigate growth dynamics by fitting multiple growth models (von Bertalanffy, Gompertz, Logistic, and Richards) to length-at-age data; (3) evaluate how gonadal development, a direct proxy for reproductive investment [10], shapes sex-specific growth and reproductive strategies; and (4) apply Generalized Linear Models to quantify the impact of spatial variation on these growth patterns.

The timing of our study is particularly relevant, coinciding with the fourth year of the Yangtze River fishing moratorium. While baseline data from the moratorium’s onset would have been ideal for assessing conservation impact, this mid-term assessment provides valuable insights into early recovery patterns under reduced fishing pressure, though pre-moratorium comparisons are limited by historical data gaps. Nevertheless, this study provides a valuable ecological opportunity to observe initial ecological responses and establish comprehensive baseline data for future evaluations of conservation policy effectiveness. The findings from this basin-scale assessment will inform effective monitoring and management strategies within the Yangtze River ecosystem, contributing to evidence-based conservation approaches and ultimately supporting the sustainable recovery of *C. nasus* populations and the overall ecological health of this critical riverine system.

## 2. Material and Method

### 2.1. Sample Collection

**Figure 1:**
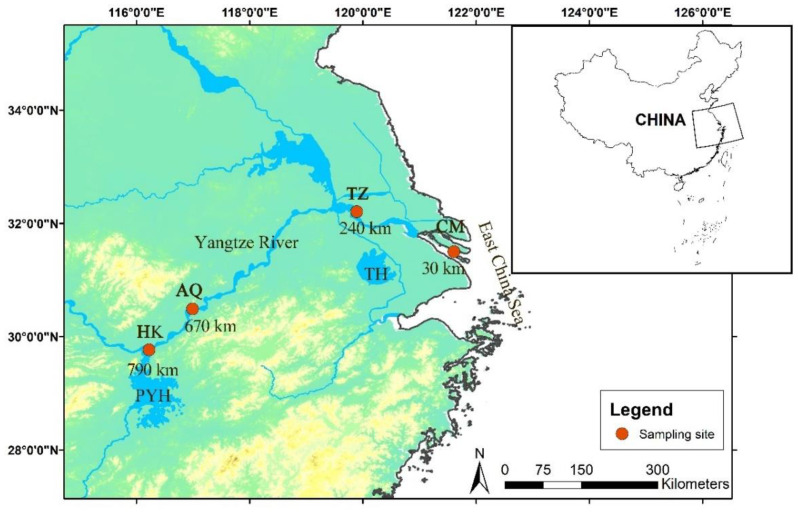
Sampling sites for Coilia nasus in the middle and lower Yangtze River, China. CM: Chongming; TZ: Taizhou; AQ: Anqing; TH: Taihu Lake; PYH: Poyanghu; HK: Hukou. The numbers indicate the distance (in river kilometers) from the sampling site to the river’s estuary.

Sampling was conducted from 30 May to 3 June 2024, during the spawning migration period of *C. nasus*. Scientific fishing permits were obtained from the provincial fishery authorities in compliance with the current Yangtze River fishing ban (2021–2031). Four sampling sites were established along the middle and lower reaches of the Yangtze River: Chongming (CM; 31°29′52″ N, 121°36′36″ E, 30 km from the estuary, Shanghai), Taizhou (TZ; 32°12′14″ N, 119°53′40″ E, 240 km, Jiangsu), Anqing (AQ; 30°29′11″ N, 116°59′39″ E, 670 km, Anhui), and Hukou (HK; 29°45′45″ N, 116°13′40″ E, 790 km, Jiangxi) (Figure 1). One sampling site was selected in each province to comply with the scientific fishing permit requirements. Specimens were collected simultaneously at all sites using one traditional drift gill net per site (40 mm mesh size) operated by experienced local fishermen. To contextualize the observed biological patterns, water temperature was recorded daily at each site using a WTW Multi 3430 multi-parameter water quality analyzer (WTW GmbH, Weilheim, Germany). Water temperatures across the sampling period ranged from 20.1 to 23.9 °C (Table S1).

Both standard length (SL) and total length (TL) were measured to the nearest 0.01 cm, and body weight (W) was recorded to the nearest 0.1 g for all specimens immediately after collection. Species identification was based on traditional morphological characteristics, particularly the diagnostic supermaxilla-to-head length ratio (>1.0, measured using precision calipers with an accuracy of 0.02 mm), confirming the specimens as the long-maxilla form of *C. nasus* [3]. The timing and location of sampling in the main channel during the established migration period supported the identification of samples as the migratory form of this species. A total of 1119 specimens was collected across all sites.

Following the morphometric assessment, all specimens were dissected for sex determination and maturity assessment. Sex and maturity were assessed based on macroscopic gonadal traits, and were classified into six maturity stages (I–VI) according to a standardized scale of morphological characteristics and coloration [18,19]. These macroscopic staging criteria have been previously validated against histological analyses specifically for *C. nasus*, demonstrating high accuracy and reliability for maturity assessment in anadromous populations [20].

### 2.2. Age Determination

For age determination, sagittal otoliths and 5–10 scales per specimen were extracted from the region posterior to the dorsal fin origin and superior to the lateral line. Scale preparation involved cleaning with distilled water and mounting on microscope slides.

Age determination was based on counting the number of opaque zones in otoliths and narrow bands in scales, which form during slow growth periods [13]. Two experienced readers conducted independent age assessments using a Zeiss Axio Lab.A1 optical microscope (Carl Zeiss Microscopy GmbH, Jena, Germany), with the readers blind to specimen lengths. Ages were accepted when both readers achieved agreement. When discrepancies occurred, age validation was performed through otolith examination. The otolith aging process involved independent counts of opaque zones by two readers, with ages accepted only upon mutual agreement.

Initial agreement between the readers on scale-based ages was 99.0%. All discrepancies (n = 11) were subsequently resolved via otolith cross-validation, yielding a 100% final consensus.

### 2.3. Length-Weight Relationship

The relationship between body weight (W) and standard length (*SL*) of *C. nasus* was estimated using the power function equation $W=a\times SL^{b}$, where *a* is the coefficient of proportionality, and *b* is the allometric growth exponent, with *b* = 3 indicating isometric growth [21]. A one-sample *t*-test was used to determine whether *b* significantly deviated from 3. Sex-specific differences in length-weight relationships were examined using analysis of covariance (ANCOVA), with *SL* as the covariate and W as the dependent variable, to compare slopes and intercepts between males and females.

### 2.4. Growth

To identify the optimal growth model for *C. nasus*, we implemented an information-theoretic multi-model inference approach [22]. Age-at-length data were fitted to four well-established growth models for teleosts: the von Bertalanffy Growth Model (VBGM) [23], the Gompertz model [24], the Logistic model [25], and the Richards model [26].

The VBGM is described by the equation: $L_{s}=L_{\infty }\left(1-e^{-k(t-t_{0})}\right)$, where *L*_s_ denotes the standard length at age *t*, *L*_∞_ represents the asymptotic length, *k* is the growth rate parameter (year^−1^), and *t*_0_ indicates the theoretical age at zero length.

The Gompertz and Logistic models, both three-parameter sigmoid curves, account for exponentially declining growth rates with increasing body size, effectively describing slow early-life growth [27]. These models are expressed as: Gompertz: $L_{s}=L_{\infty }e^{-e^{-k(t-t_{0})}}$; Logistic: $L_{s}=L_{\infty }{\left(1+e^{-k\left(t-t_{0}\right)}\right)}^{-1}$. In these equations, *L*_∞_ is asymptotic length, *k* is the growth rate parameter (year^−1^), and *t*_0_ is the age at the curve’s inflection point.

Additionally, the Richards model, a four-parameter sigmoid model that generalizes the VBGM [28], is expressed as: $L_{s}=L_{\infty }{\left[1+\left(\frac{1}{\rho }\right)e^{-k\left(t-t_{0}\right)}\right]}^{-\rho }$, where *L*_∞_, *k*, and *t*_0_ are as defined above, and *ρ* is a shape parameter controlling the growth curve.

All four candidate models were fitted to *C. nasus* length-at-age data using nonlinear least squares in R version 4.4.1 [29]. The relative support for each model was evaluated using Akaike’s Information Criterion for small sample sizes (AICc) [30]. Models with an AICc value within two units of the calculated value for the best approximating model (lowest AICc) were considered to have substantial empirical support, while larger differences indicated considerably less support [30]. The Akaike weight (*w_i_*) of each model *i* was calculated to quantify the plausibility of each model, given the data and the set of four models, using: $w_{i}=\frac{e^{-0.5\Delta_{i}}}{\sum_{i=4}^{4}e^{-0.5\Delta_{i}}}$, where $\Delta_{i}=AIC_{c,\min}-AIC_{c,i}$. The Akaike weight represents the weight of evidence in favor of model *i* being the actual best model of the available set of models [22].

Support for sex-specific growth curves was evaluated by comparing the AICc of the best-fitting model for the pooled dataset to the sum of the AICc values from the same model fitted separately to female and male data [31]. This comparison provided substantial support for separate growth curves for females and males.

Based on these results, separate growth models for females and males were used to evaluate the spatial variation in *C. nasus* growth across the estuary distance gradient. We employed generalized linear models (GLMs) to analyze the residuals from sex-specific growth models in relation to estuary distance. Both linear relationships and nonlinear relationships using cubic splines (2–3 degrees of freedom) were examined, with AICc used to determine the optimal functional form.

### 2.5. Statistical Analysis

Due to the non-normal distribution of variables (standard length, body weight, gonadal maturity stages, and age; Shapiro–Wilk test, all *p*-values < 0.05), non-parametric statistical tests were applied. The Kruskal–Wallis test was used to assess differences in standard length, body weight, gonadal maturity stages, and age across sampling sites and between sexes. For pairwise comparisons following the Kruskal–Wallis test, the Wilcoxon rank sum test (equivalent to the Mann–Whitney U test in R) was employed, with *p*-values adjusted using the Bonferroni correction. The chi-square (*χ*^2^) test was used to analyze differences in sex distribution across sampling sites. All statistical analyses were performed using R software (version 4.4.1).

## 3. Result

### 3.1. Sex Ratio and Gonadal Maturity Patterns

**Figure 2:**
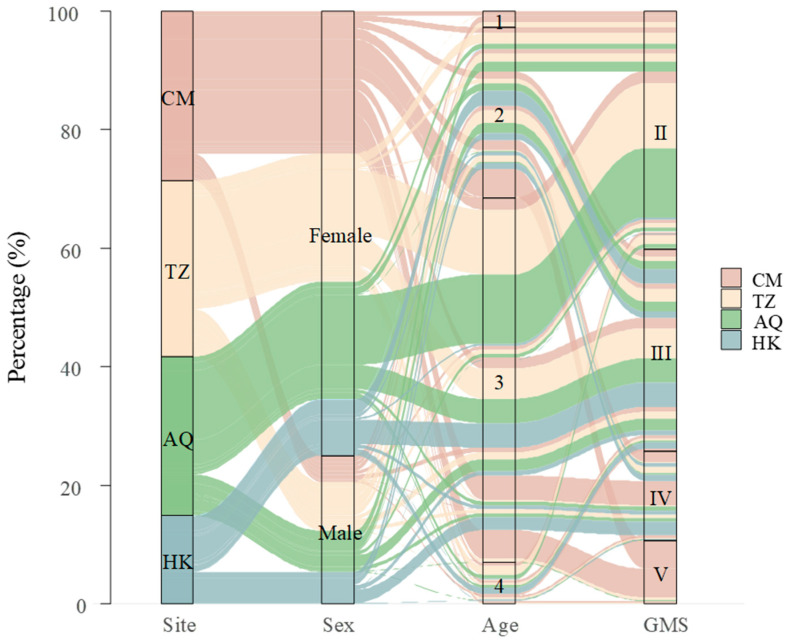
The interrelationships among sex, age, and gonadal maturity stages of *Coilia nasus* in the middle and lower Yangtze River, China.

The *C. nasus* population exhibited a significant female bias, with females comprising 75.0% (*n* = 839) and males 25.0% (*n* = 280) of the sample. The overall sex ratio (female–male) was 3.00:1, which deviated significantly from the expected 1:1 ratio (*χ*^2^ test, *χ*^2^ = 279.25, d.f. (degrees of freedom) = 1, *p* < 0.001). The sex ratio varied significantly across sampling sites (*χ*^2^ test, *χ*^2^ = 26.12, d.f. = 3, *p* < 0.001), showing a downstream-upstream gradient (Figure 2). The highest female–male ratio occurred at the estuarine CM site (5.27:1), followed by AQ (2.85:1) and TZ (2.66:1), with the lowest ratio observed at the uppermost HK site (1.77:1; Figure 3).

Analysis of gonadal maturity stages (GMS) revealed no significant differences between sexes (Wilcoxon rank sum test, *W* = 110,076, *p* = 0.09), but showed highly significant spatial variation across sampling sites (Kruskal–Wallis test, *χ*^2^ = 278.35, d.f. = 3, *p* < 0.001). Post hoc Dunn’s tests with Bonferroni correction revealed two distinct site groupings: AQ-TZ (*p* = 0.59) and CM-HK (*p* = 0.38), with highly significant differences between all other site pairs (*p* < 0.001).

**Figure 4:**
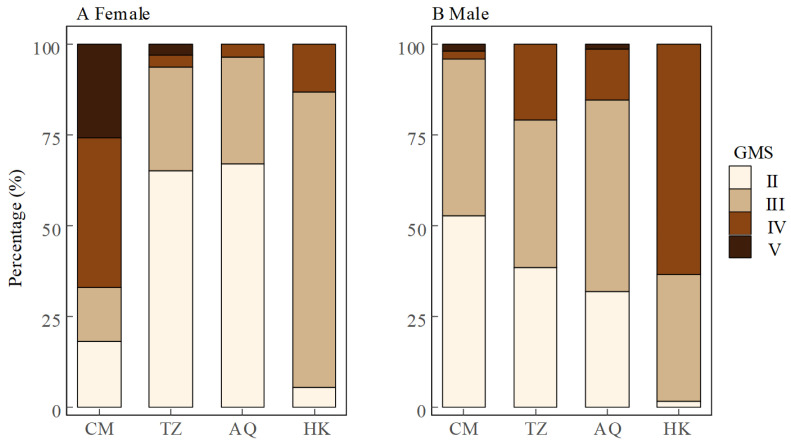
Percentage of gonadal maturity stages (GMS) in female (**A**) and male (**B**) *Coilia nasus* in the middle and lower Yangtze River, China.

Spatial patterns in gonadal maturity showed sex-specific trends. At the estuarine site (CM), females displayed advanced gonadal maturity with 66.9% in stages IV and V, while males remained predominantly (96.1%) in earlier stages II and III. Conversely, at the uppermost site (HK), males exhibited advanced maturity (63.3% in stage IV) while females were primarily (81.1%) in stage III. The intermediate sites (TZ and AQ) showed similar patterns, with females predominantly in stage II (65.3% and 67.1%, respectively) and males distributed across stages II and III, showing modest progression toward advanced stages (Figure 4). Overall, female GMS exhibited a U-shaped distribution across sites (CM > HK > TZ > AQ), whereas male GMS showed a consistent upstream increase (CM < TZ ≈ AQ < HK) (Figure 2). Notably, the spatial variation in GMS was more pronounced in females (Kruskal–Wallis test, *χ*^2^ = 310.04, d.f. = 3, *p* < 0.001) than in males (*χ*^2^ = 66.52, d.f. = 3, *p* < 0.001), indicating that geographical factors exert a stronger influence on female gonadal maturity.

### 3.2. Age Composition

The age ranged from 1 to 4 years, with the majority being 3-year-olds (61.6%, *n* = 689), followed by 2-year-olds (28.7%, *n* = 321), 4-year-olds (7.0%, *n* = 78), and 1-year-olds (2.8%, *n* = 31, Figure 2). Females (*n* = 839) were significantly older than males (*n* = 280), with mean ages of 2.84 ± 0.57 and 2.39 ± 0.67 years, respectively (Wilcoxon rank sum test, *W* = 158,563, *p* < 0.001). Age distributions varied significantly across sampling sites for the overall population and both sexes (Kruskal–Wallis tests: overall, *p* < 0.001; females, *p* < 0.001; males, *p* = 0.0012).

**Figure 5:**
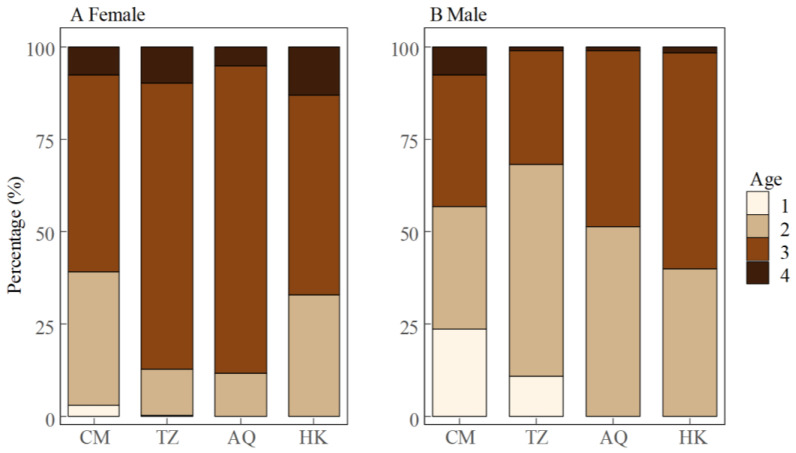
Age composition of female (**A**) and male (**B**) *Coilia nasus* in the middle and lower Yangtze River, China.

The age structure showed distinct spatial and sex-specific patterns (Figure 5). Among females, age group 3 dominated all sampling sites, particularly at AQ (82.9%) and TZ (77.3%), representing the main spawning population. The estuarine site (CM) displayed the most diverse female age structure, with substantial proportions of age group 2 (36.1%) and age group 4 (7.8%). Males showed a more balanced distribution between age groups 2 and 3. This pattern was most distinct at the CM site, where males were distributed across three age groups, with comparable proportions of age 1 (23.5%), age 2 (33.3%), and age 3 (35.3%). With increasing distance from the estuary (TZ → AQ → HK), the age structure of males shifted toward older individuals, characterized by a decreasing proportion of 2-year-olds (TZ: 57.1%, AQ: 51.3%, HK: 40.0%) and an increasing proportion of 3-year-olds (TZ: 30.8%, AQ: 47.4%, HK: 58.3%), with this trend being most prominent at HK. Notably, age group 1 occurred only at CM and TZ sites, being sparse in females (CM: 3.0%, TZ: 0.4%) but substantial in males (CM: 23.5%, TZ: 11.0%).

### 3.3. Standard Length and Body Weight

Morphometric measurements were recorded for all 1119 specimens. Total length (TL) ranged from 18.98 to 40.73 cm (mean ± SD = 32.19 ± 3.63 cm), and standard length (SL) ranged from 16.93 to 38.00 cm (mean ± SD = 29.81 ± 3.44 cm). Body weight varied from 16.4 to 218.3 g (mean ± SD = 96.4 ± 32.0 g). Females were significantly larger than males in both standard length and body weight, as determined by the Wilcoxon rank sum test (*W* = 171,670 for standard length, *W* = 181,976 for body weight, both *p* < 0.001). The mean standard length and body weight for females were 30.44 ± 3.23 (±SD) cm and 103.4 ± 30.8 (±SD) g, respectively, compared to 27.92 ± 3.36 cm and 75.4 ± 25.8 g for males.

**Figure 6:**
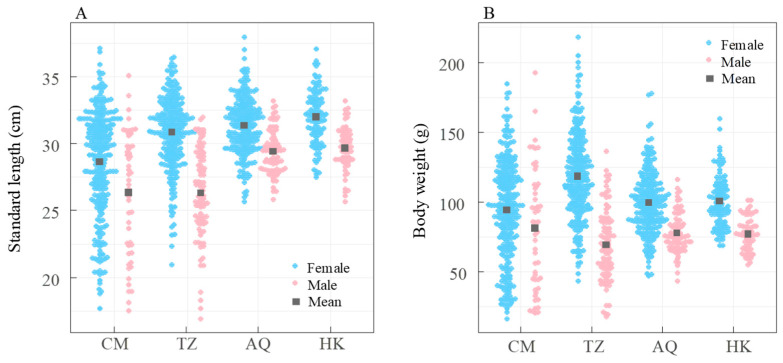
Site-specific variations in standard length (**A**) and body weight (**B**) of *Coilia nasus* in the middle and lower Yangtze River, China. Blue circles: females; red solid circles: males; gray squares: mean values.

Kruskal–Wallis analysis revealed significant site-specific and sex-based differences in both standard length and body weight, with females consistently larger than males across all sites (all *p* < 0.05). Standard lengths for both sexes generally increased with distance from the estuary, although body weight trends were less consistent (Figure 6). Females at TZ were significantly heavier than those at AQ and HK (both *p* < 0.001), between which no significant difference was observed (*p* = 0.75). For males, body weights at HK and AQ were not significantly different (*p* = 0.97) but were lower than at TZ. At the AQ and HK sites, all individuals exceeded 25 cm in standard length, whereas 17.8% of females and 39.2% of males at CM, and 3.7% of females and 34.1% of males at TZ, were below 25 cm (Figure 6A).

**Figure 7:**
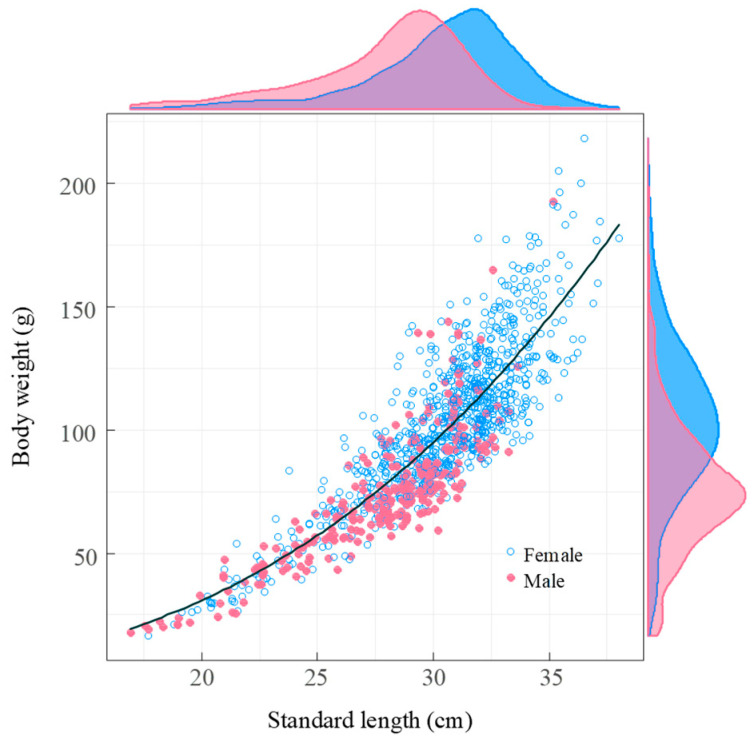
The relationship between standard length and body weight of *Coilia nasus* in the middle and lower Yangtze River, China. The scatter plot utilizes blue solid circles for females and pink solid circles for males, showcasing their respective distributions. The black line represents the fitted regression line. The density distributions along the axes highlight the differences between the sexes, with a blue density curve for females and a pink density curve for males.

The length-weight relationship analysis using ANCOVA revealed that the relationship between standard length and body weight did not differ significantly between males and females in our study area (*F* = 0.16, d.f. = 1, *p* = 0.69) allowing for data to be pooled across sexes. The estimated parameters for the length-body weight relationship were *a* = 0.0076 (95% confidence limits: 0.0052–0.0111) and *b* = 2.77 (95% C.L.: 2.66–2.88). A one-sample *t*-test confirmed a negatively allometric growth pattern (*b* < 3, *t* = −4.074, d.f. = 1117, *p* < 0.001), where weight increases less than proportionately to length (Figure 7).

### 3.4. Growth Model Selection and Parameter Estimation

Of the four growth models evaluated, the von Bertalanffy growth model (VBGM) was identified as the best-fitting model for all individuals (*w* = 0.60), although the Gompertz model also received considerable support (ΔAICc = 1.79, *w* = 0.24). The Logistic and Richards models were significantly less supported, with ΔAICc values exceeding 3.8. Our analyses strongly favored sex-specific growth models, as evidenced by a substantial AICc difference of 33.31 between the VBGM fitted to all data (AICc = 1728.97) and the sum of the AICc values from fitting the same model to female and male data separately (AICc = 1337.27 + 358.39 = 1695.66) (Table 1). This significant difference indicated that sex-specific modeling was more appropriate for this species.

**Figure 8:**
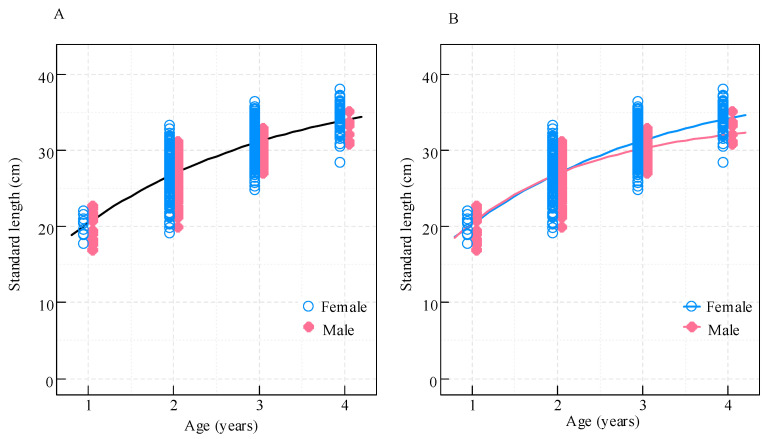
Von Bertalanffy growth curves for *Coilia nasus* in the middle and lower Yangtze River, China. (**A**) Pooled sexes; (**B**) Sex-specific models (pink: males; blue: females).

The VB growth curves for females and males were nearly identical until age 2. However, females showed greater length-at-age thereafter, with the difference increasing over time (Figure 8). Although males had a larger growth rate parameter (*k*), females had a greater predicted mean asymptotic length (*L_∞_*), exceeding that of males by 5.67 cm (Table 1).

**Figure 9:**
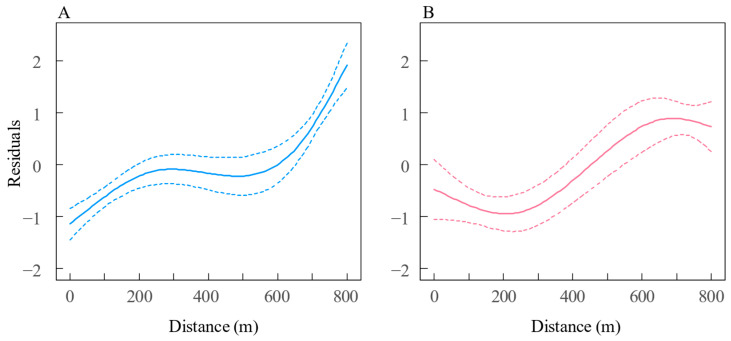
Predicted residual standard-length trends relative to estuary distance for (**A**) female and (**B**) male *Coilia nasus* in the middle and lower Yangtze River, China. Solid lines: GLM predictions with cubic splines (d.f. = 3); dashed lines: ±2 SD.

Spatial variation in growth was investigated using GLM models, which revealed that the residual length-at-age from the VBGM for both sexes varied significantly with estuary distance. This relationship was best captured by a cubic spline with three degrees of freedom in the GLM, as indicated by the lowest AICc values (Table 2). For both sexes, a strong relationship was observed between residuals and estuary distance: female residuals consistently increased with distance, while male residuals initially decreased until approximately 240 km (TZ) before increasing (Figure 9).

## 4. Discussion

### 4.1. Sexual Size Dimorphism and Growth Patterns

Our study revealed significant sexual size dimorphism in *C. nasus*, with females exhibiting greater body lengths and older ages compared to males. Specifically, females averaged 30.44 ± 3.23 cm in standard length and 2.84 years in age, whereas males averaged 27.92 ± 3.36 cm and 2.39 years, respectively. The von Bertalanffy growth parameters provided quantitative evidence of this dimorphism, with males showing a higher growth coefficient (*k*) but lower asymptotic length (*L*_∞_), while females displayed the opposite pattern. These sex-specific differences are consistent with patterns documented in previous research on fish species [32,33,34].

These morphometric and growth parameter differences can be interpreted as distinct reproductive strategies between sexes. Female *C. nasus* employ a size-maximization strategy, delaying sexual maturity to achieve larger body size, which directly enhances reproductive capacity and is reflected in their higher *L*_∞_ values. This adaptive strategy is supported by Song et al. (2022), who demonstrated that fecundity in mature females ranges from 29,908 to 74,041 eggs, with a strong positive correlation with body size [16]. In contrast, male *C. nasus* adopt a time-minimization strategy, prioritizing early maturation over maximum growth potential—a pattern commonly observed in species without paternal care [35,36,37].

The age distribution analysis provides additional evidence for these contrasting reproductive strategies. The predominance of 3-year-old females across sampling sites, particularly at AQ (82.9%) and TZ (77.3%), indicates that females typically require three years to achieve the optimal body size for reproduction—consistent with their size-maximization strategy. Males, however, displayed a more heterogeneous age structure, especially at the estuarine CM site, with substantial proportions of age-1 (23.5%), age-2 (33.3%), and age-3 (35.3%) individuals. This participation of younger males in reproductive migration directly reflects their time-minimization strategy. The faster early growth rate (higher *k*) in males facilitates earlier maturity and increased mating opportunities, while the larger asymptotic length (*L*_∞_) in females maximizes fecundity.

### 4.2. Spatial Patterns in Size Distribution: Evidence for Size-Dependent Migration Strategy

Building upon our findings on sexual dimorphism, we identified a distinct spatial gradient in *C. nasus* body size, characterized by progressive increases in length with greater distance from the estuary. This pattern was consistent across sexes, with females maintaining their size advantage at all sampling sites (Figure 6). Based on the documented migration speed of *C. nasus* (approximately 23 km/day) [7] and the distances to our sampling locations, we can estimate that migration initiation likely varied by location, from mid-April (HK) to late May (CM). This spatial variation aligns with previous observations of seasonal decreases in size among estuarine *C. nasus* populations [16]. This size-dependent migration pattern is consistent with findings by Li et al. (2007), who reported that spawning females in the Yangtze estuary were significantly smaller than those in upstream spawning grounds [20].

Our GLM analysis provided statistical confirmation of this pattern, revealing a significant positive relationship between standard length residuals and distance from the estuary for both sexes. This finding represents a specific form of size-dependent migration, similar to patterns observed in Norwegian spring-spawning herring (*Clupea harengus*) where “the distance of spawning migration tends to increase with the length and condition of the fish” [38]. The physiological basis for this pattern likely relates to energetics, as the relative energy cost of migration decreases markedly with increasing body size [39], and smaller individuals generally experience higher rates of tissue depletion and energy loss during long-distance movement [40,41,42].

Field data strongly support this conclusion: all individuals at the distant AQ and HK sites exceeded 25 cm in standard length, whereas substantial proportions of the populations at CM (17.8% of females, 39.2% of males) and TZ (3.7% of females, 34.1% of males) were below this threshold (Figure 6A). This pattern likely reflects an evolutionary trade-off in migration decisions. Larger fish experience lower relative energetic costs during migration [38,43], making upstream spawning grounds accessible only to individuals with sufficient size and energy reserves.

Similar size-dependent migratory patterns represent an evolutionarily convergent adaptation documented across various migratory fish species, including American smelt (*Osmerus mordax*) [44], Atlantic salmon (*Salmo salar*), and anadromous brown trout (*Salmo trutta*) [43]. From a conservation perspective, this size-dependent migration pattern has significant implications. Historically, *C. nasus* was reported to migrate as far upstream as Dongting Lake, but contemporary migrations rarely extend beyond Poyang Lake [20]. This reduction in migratory range likely results from population decline and the loss of larger individuals capable of long-distance migration. However, recent evidence suggests that protection measures can reverse this trend. Jiang et al. (2023) [45] documented the return of anadromous *C. nasus* to the Xiangjiang River in Hunan Province following the implementation of a ten-year fishing ban in the Yangtze River. Otolith microchemistry analysis confirmed that these individuals had migrated from estuarine waters to this historically important but long-abandoned habitat. As observed in other migratory species, changes in population size structure can significantly alter migration patterns and habitat use [38,43]. Since fecundity increases significantly with body size, the protection of these larger individuals that reach distant upstream spawning grounds is particularly crucial for population recruitment and recovery.

### 4.3. Gonadal Maturity Patterns: Evidence for Sex-Specific Reproductive Strategies

Our analyses revealed distinct spatial patterns in gonadal maturity stages (GMS) between sexes. At the estuarine site (CM), females exhibited advanced gonadal development with 66.9% in stages IV–V, while males remained predominantly (96.1%) in earlier stages II–III. Conversely, at the uppermost site (HK), males showed more advanced maturity (63.3% in stage IV) while females were primarily (81.1%) in stage III. This contrasting pattern suggests sex-specific reproductive timing strategies during upstream migration.

The observed spatial variation in gonadal development aligns with previous findings by Li et al. (2007) [20] on this species, where the proportion of mature females varied significantly by location and sampling period. They documented that during April-June, the percentage of mature females was consistently higher in the estuary than at upstream locations, with maturation patterns showing temporal progression. This consistent pattern across studies suggests a well-established reproductive strategy in *C. nasus*, where gonadal maturation is carefully timed to coincide with arrival at specific spawning locations.

Water temperature likely plays a crucial role in this pattern. At the Yangtze River estuary, water temperatures increase from 15.0–17.0 °C in mid-April to 21.8–27.2 °C by late May [15,16], which would accelerate gonadal development for individuals arriving later. This timing difference explains why females at CM (later migrants) showed more advanced gonadal development than those at upstream sites, who began migration earlier under colder conditions.

## 5. Conclusions

This study revealed significant sexual dimorphism and spatial patterns in *C. nasus* population structure along the middle-lower Yangtze River during the 2024 spawning season. Female *C. nasus* exhibited greater body lengths and ages than males, with contrasting growth patterns where males showed higher growth coefficients but lower asymptotic lengths. Our findings suggest a pronounced size-dependent migration strategy, with larger individuals migrating earlier and farther upstream—a pattern statistically validated by GLM analysis showing significant positive relationships between body length residuals and distance from the estuary. Based on these findings of size-dependent upstream migration and sex-specific life-history traits, we recommend several targeted management actions derived directly from our data. First, implement spatial management strategies to protect critical spawning habitats for larger individuals in upstream reaches, particularly during peak migration periods. Second, develop adaptive fishing regulations that account for sexual dimorphism in growth patterns and reproductive timing. Third, prioritize habitat connectivity restoration to facilitate natural migration pathways. Fourth, enhance seasonal protection measures during reproductive periods when spatial segregation is most pronounced. These science-based recommendations provide an essential framework for the long-term recovery of this ecologically important anadromous species.

Our study had several limitations, including restricted sampling points due to fishing regulations, particularly the large gap between TZ and AQ; the absence of fecundity and GSI measurements due to field sampling constraints; and the limited one-week sampling period, which prevented analysis of seasonal variations. Given these temporal and spatial constraints, our research provides valuable mid-term baseline data during the fourth year of the ten-year fishing ban in the Yangtze River (2021–2031). Despite the absence of pre-moratorium baseline data, our study offers the first systematic analysis of spatial and sex-specific growth variations in *C. nasus* populations under reduced fishing pressure. These baseline findings, collected during the fourth year of the fishing moratorium, provide important reference data for evaluating conservation effectiveness and developing management strategies for this endangered species. Future research should incorporate multi-year sampling designs, fecundity and GSI assessments, and otolith microchemistry analysis to further investigate life history patterns and migration pathways, and assess long-term population recovery trends.

## Figures and Tables

**Figure 3:**
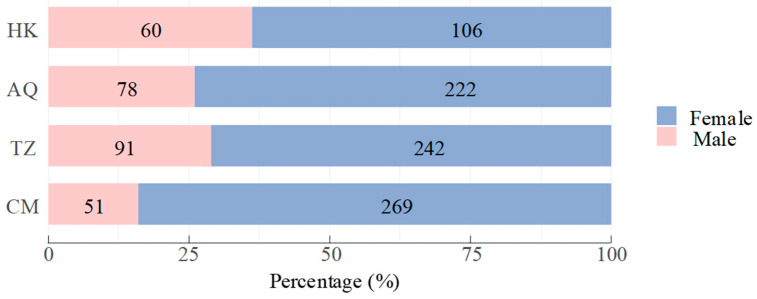
Sex ratio composition of *Coilia nasus* populations in the middle and lower Yangtze River, China. Numbers on bars indicate sample sizes for each sex.

**Table 1:** Parameter estimates (±standard error) from four candidate growth models for *Coilia nasus* in the middle and lower Yangtze River, China.

Sex	Model	*L_∞_* (cm)	*K* (Year^−1^)	*t*_0_ (Year)	*ρ*	AICc	ΔAICc	*w_i_*
Pooled sexes	**VBGM**	**39.36 (1.36)**	**0.42 (0.05)**	**−0.76 (0.18)**		**1728.97**	**0.00**	**0.60**
	Gompertz	37.62 (0.97)	0.58 (0.06)	0.13 (0.07)		1730.76	1.79	0.24
	Logistic	36.59 (0.78)	0.74 (0.06)	0.64 (0.05)		1733.18	4.21	0.07
	Richards	37.61 (2.53)	0.58 (0.29)	0.14 (1.10)	100.00 (168.99)	1732.79	3.82	0.09
Female	**VBGM**	**39.68 (1.80)**	**0.42 (0.07)**	**−0.71 (0.26)**		**1337.27**	**0.00**	**0.43**
	Gompertz	38.14 (1.33)	0.57 (0.08)	0.16 (0.11)		1338.02	0.75	0.29
	Logistic	37.24 (1.09)	0.71 (0.08)	0.67 (0.07)		1339.08	1.81	0.17
	Richards	38.13 (3.11)	0.57 (0.36)	0.16 (1.47)	100.00 (227.14)	1340.05	2.78	0.11
Male	**VBGM**	**34.01 (1.03)**	**0.65 (0.10)**	**−0.42 (0.18)**		**358.39**	**0.00**	**0.39**
	Gompertz	33.22 (0.79)	0.83 (0.10)	0.13 (0.10)		358.96	0.57	0.29
	Logistic	32.65 (0.64)	1.02 (0.11)	0.49 (0.07)		359.62	1.23	0.21
	Richards	33.21 (2.94)	0.83 (0.81)	0.14 (1.89)	100.00 (430.62)	361.02	2.63	0.11

Notes: AICc is the Corrected Akaike Information Criterion, ΔAICc is the difference in Corrected Akaike Information Criterion, *w_i_* is the Akaike weight, and *L_∞_*, *k*, *t*_0_, *ρ* are parameters of the growth models. Bold values indicate the best-fitting model based on the lowest AICc value.

**Table 2:** Parameter estimates from generalized linear models (GLM) for *Coilia nasus* in the middle and lower Yangtze River, China.

Sex	Distance Effect	Model Expression	AICc	ΔAICc	*w_i_*
Female	Linear	*R* = *α* × *dis* + *ε*	3597.93	16.82	0.00
	Cubic spline (d.f. = 2)	*R* = *spline*_2_ (*dis*) + *ε*	3599.94	18.83	0.00
	Cubic spline (d.f. = 3)	*R* = *spline*_3_ (*dis*) + *ε*	**3581.11**	**0.00**	**1.00**
Male	Linear	*R* = *α* × *dis* + *ε*	1104.74	7.69	0.02
	Cubic spline (d.f. = 2)	*R* = *spline*_2_ (*dis*) + *ε*	1101.79	4.75	0.08
	Cubic spline (d.f. = 3)	*R* = *spline*_3_ (*dis*) + *ε*	**1097.05**	**0.00**	**0.90**

Notes: *R* denotes the residual standard length, *α* indicates the coefficient for the distance effect (*dis*), spline_2_ and spline_3_ represent cubic spline functions with 2 and 3 degrees of freedom respectively, and *ε* is the error term. AICc refers to the corrected Akaike Information Criterion, ΔAICc represents the difference between the AICc of each candidate model and that of the best model, and *w_i_* denotes the Akaike weight. d.f. stands for degrees of freedom. Bold values indicate the best-supported models based on the lowest AICc values.

## Data Availability

The data supporting the findings of this study are available from the corresponding author, Kai Liu (liuk@ffrc.cn), upon reasonable request.

## Supplementary Materials

The following supporting information can be downloaded at https://www.mdpi.com/article/10.3390/biology14091211/s1, Table S1: Water Temperature (°C) during *Coilia nasus* sampling in the middle and lower Yangtze River, China.
